# An area and power efficient ternary serial adder using phase composite ZnO stack channel FETs†

**DOI:** 10.1039/d5na00045a

**Published:** 2025-04-08

**Authors:** Kiyung Kim, Sunmean Kim, So-Young Kim, Yongsu Lee, Hae-Won Lee, Seokhyeong Kang, Byoung Hun Lee

**Affiliations:** a Department of Electrical Engineering, Pohang University of Science and Technology Cheongam-ro 77, Nam-gu Pohang Gyeongbuk 37673 Republic of Korea bhlee1@postech.ac.kr; b School of Electronic and Electrical Engineering, Kyungpook National University Daegu 41556 Republic of Korea

## Abstract

Multi-valued logic is the subject of ongoing investigation owing to its potential to reduce the complexity of logic circuits and interconnect lengths, thereby reducing system power consumption. In this work, ternary stack channel field-effect transistors (SCFETs) are used as unit devices to realize multi-valued logic. The thickness of each ZnO layer in the SCFET is modulated to obtain the device parameters to control the intermediate-state range and saturation current. Using the experimental results, ternary circuits are modeled and simulated to demonstrate that the unique characteristics of SCFETs can be utilized in designing a ternary full adder. The designed ternary full adder requires only 12 devices (approximately 29% of the binary full adder device count). The ternary serial adder has a competitive power-delay product value of approximately 7 fJ at *V*_DD_ = 1 V and an effective oxide thickness of 1 nm. These results indicate that SCFET-based ternary circuits are a promising alternative for extremely low-power applications.

## Abstract 2

This paper proposes a highly efficient ternary serial adder (TSA) in terms of area and power. The unique characteristics of phase composite ZnO stacked channel field-effect transistors are exploited for the TSA circuit.

## Graphical abstract

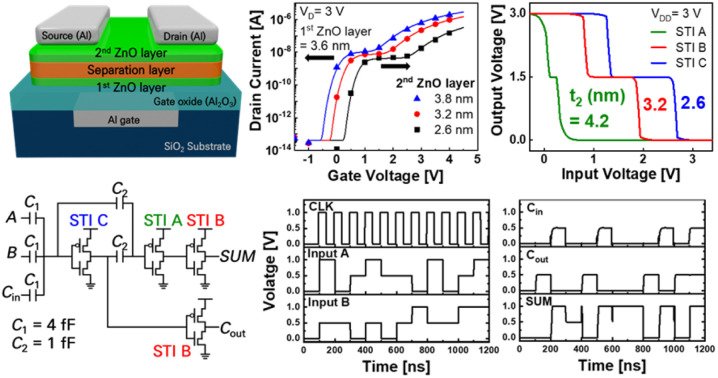

## Introduction

In recent years, the physical scaling limits of complementary metal-oxide-semiconductor (CMOS) technology have become evident, and numerous workarounds, such as using backside power distribution networks and monolithic and heterogeneous three-dimensional (3D) integration, are being actively pursued.^1^ In addition, more disruptive technologies, such as quantum computing, analog computing, and multi-valued logic (MVL), are receiving increasing attention.^2^ Recent studies on MVL technology primarily aim to realize lower power consumption at the system level by reducing the circuit complexity and interconnect length.^3,4^

However, replacing an entire binary logic system with an MVL system remains challenging. The most considerable obstacle is the absence of logic design fundamentals, such as Boolean logic. Notably, several device studies feature ternary inverters as an application, despite the lack of comprehensive design methodologies for more high-level logic circuits, such as NMIN, NMAX, and ADDER. Although some proposals incorporate advanced ternary architectures, they employ the conventional binary device model, which tends to increase the complexity of circuit design. This approach is contrary to the underlying motivation of MVL technology. Therefore, a suitable circuit design method and proper unit MVL devices are essential for the practical realization of ternary systems. Moreover, the unit MVL device should be compatible with the CMOS integration process and can be fabricated using low-thermal-budget integration processes, considering the potential co-integration with CMOS devices or monolithic 3D integration in the back-end-of-line structure.

Thus far, numerous devices have been proposed for MVL applications,^5,6^ including various heterojunction devices exhibiting negative differential transconductance (NDT),^7–12^ quantum dot gate field-effect transistors (FETs),^13–15^ carbon nanotube FETs (CNTFETs),^16–20^ graphene barristors^21^ and ternary CMOSs.^22–24^ Unfortunately, in most cases, the reported device development has not progressed beyond the demonstration of a ternary inverter or cannot fulfill the requirements for process feasibility and scalability.

Recently, Lee *et al.*^25^ proposed a ternary stack channel FET (SCFET), which contains two stacked nanosheet channels, for ternary logic applications. The stepwise ternary current–voltage (*I*–*V*) characteristics of the SCFET were optimized by saturating the conduction current of the first channel using a novel zero-differential transconductance (ZDT) mechanism. ZDT is a new conduction mechanism discovered in ZnO channels comprising phase composite structures, which have crystalline and amorphous structures mixed in a specific manner to yield a unique band structure, limiting the total number of carriers in the conduction band by mobility-edge quantization.^25^ In this structure, the level and range of the intermediate-state current (*I*_1_) can be modulated by adjusting the thickness of each channel.^26^ Following the demonstration of the n-type SCFET, a p-type SCFET was demonstrated by Lee *et al.* in 2023,^27^ enabling the development of a complementary ternary circuit.

Among various ternary circuit proposals, the ternary full adder (TFA) design using CNTFETs proposed by Moaiyeri *et al.*^28^ is simple and intuitive. In their design, a standard ternary inverter (STI) requires six CNTFETs. A critical limitation of this proposal is that CNTs with eight different diameters are required to implement the TFA, which makes the design infeasible. We found that the SCFET has a strong architectural synergy with Moaiyeri's TFA design because each STI can be realized with only two SCFETs, and the unique versatility of *I*_1_ modulation can be used to implement the three types of STIs more practically.

In this work, we studied the impacts of physical parameters of SCFETs having ZnO/Al-2,3-dimercapto-1-propanol (Al-DMP)/ZnO stack channels. Experimental data were systematically analyzed and reflected in the SPICE model for the SCFET. Using the newly developed device model, a ternary serial adder (TSA) was designed to minimize the power-delay product (PDP). A TSA using SCFETs was designed with a low device count of 33 and PDP of 7.15 fJ. The PDP value was one-third that of the design using the CNTFETs, confirming that SCFET-based ternary circuit technology has the potential to be a technical choice for extremely low-power applications.

## Experimental section

### Fabrication process of ZnO SCFETs

A lift-off photoresist for the buried gate was patterned using *i*-line photolithography. A 70 nm oxide trench was formed on a 300 nm-thick SiO_2_ substrate using inductively coupled plasma reactive ion etching (ICP-RIE) with Ar (30 sccm) and CF_4_ (30 sccm) plasma. The working pressure was 10 mTorr, and the process time was 110 s. The trench was filled with a 10 nm-thick Ti adhesion layer and a 60 nm-thick Al layer using electron-beam (E-beam) evaporation. Upon using the photoresist strip in the lift-off process, the buried gate was polished to remove the rabbit ear defects at the edge of the metal pattern. The Al_2_O_3_ gate dielectric layer was deposited *via* thermal ALD with a trimethyl-aluminum (TMA) precursor at 200 °C. For the uniform deposition of the channel material, the Al_2_O_3_ surface was changed from hydrophobic to hydrophilic using a UV-ozone generator. The first ZnO layer was deposited using a novel high-pressure (1.1–1.3 Torr) and long-dosing time (20 s) ALD process. The Al-DMP/Al_2_O_3_ SL and second ZnO layer were sequentially deposited using the same ALD process. Diethyl zinc (DEZ) and H_2_O sources were used for ZnO. TMA and 2,3-dimercapto-1-propanol (DMP) were used for Al-DMP. TMA and H_2_O were used for Al_2_O_3_. A 70 nm Al layer patterned by the E-beam evaporation and lift-off process was employed for the source and drain electrodes. Finally, the channel area was defined using the ICP-RIE process.

## Results & discussion

**Fig. 1:**
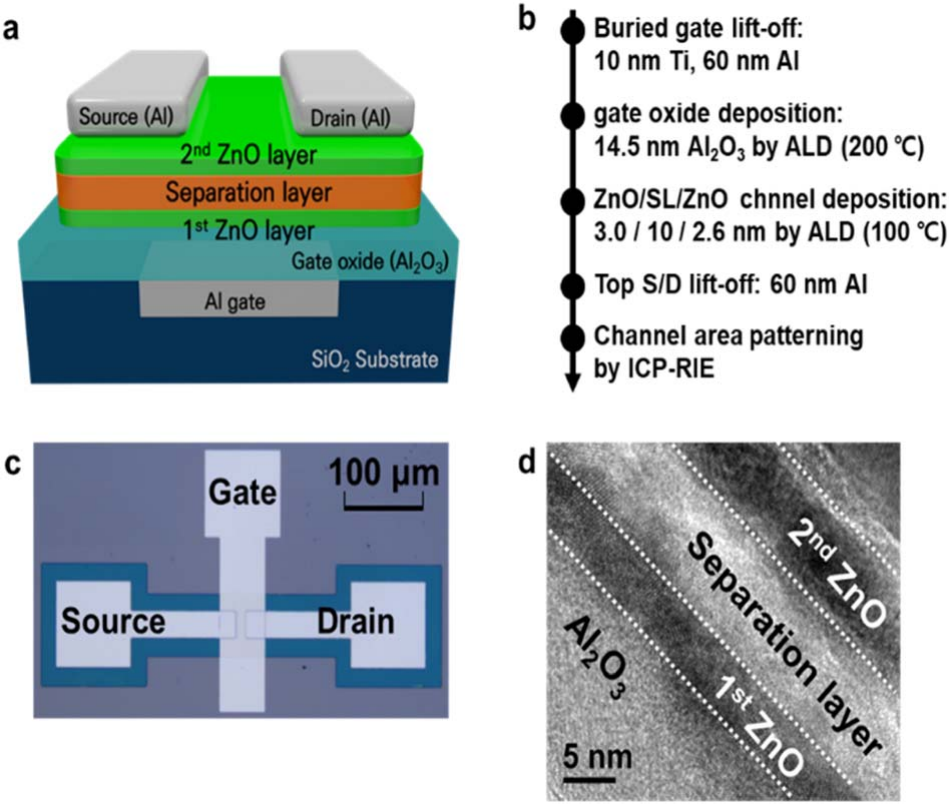
(a) Schematic structure of the ZnO/AlDMP/ZnO SCFET. (b) Fabrication process flow. (c) Top-down optical photograph of the fabricated device. (d) TEM photograph of the stacked channel structure.

A three-dimensional (3D) schematic of an SCFET is shown in Fig. 1a. In the fabrication process, the lift-off patterns for the buried gate were formed using photolithography on a 300 nm-thick SiO_2_ substrate. Subsequently, the 70 nm oxide trench was etched using inductively coupled plasma reactive ion etching (ICP-RIE). The oxide trench was filled with a 10 nm-thick Ti adhesion layer and a 60 nm-thick Al layer. Upon using the photoresist strip in the lift-off process, the buried gate was completed with a polishing process to remove the rabbit ear defects at the edge of the metal pattern. The Al_2_O_3_ gate dielectric layer was then deposited *via* thermal atomic layer deposition (ALD). The first ZnO layer, Al-DMP/Al_2_O_3_, the separation layer (SL), and the second ZnO layer were deposited in sequence using a novel high-pressure (approximately 1.3 Torr) and long-dosing time (approximately 20 s) ALD process that can form a very thin (2–4 nm) and dense film.^26^ A 70 nm Al layer patterned using the lift-off process was used as the source and drain electrodes. Finally, the channel area was defined using the ICP-RIE process. The process flow and a top-down optical image of the fabricated SCFET are shown in Fig. 1b and c, respectively. A cross-sectional transmission electron microscopy (TEM) photograph of the stacked channel is shown in Fig. 1d.

**Fig. 2:**
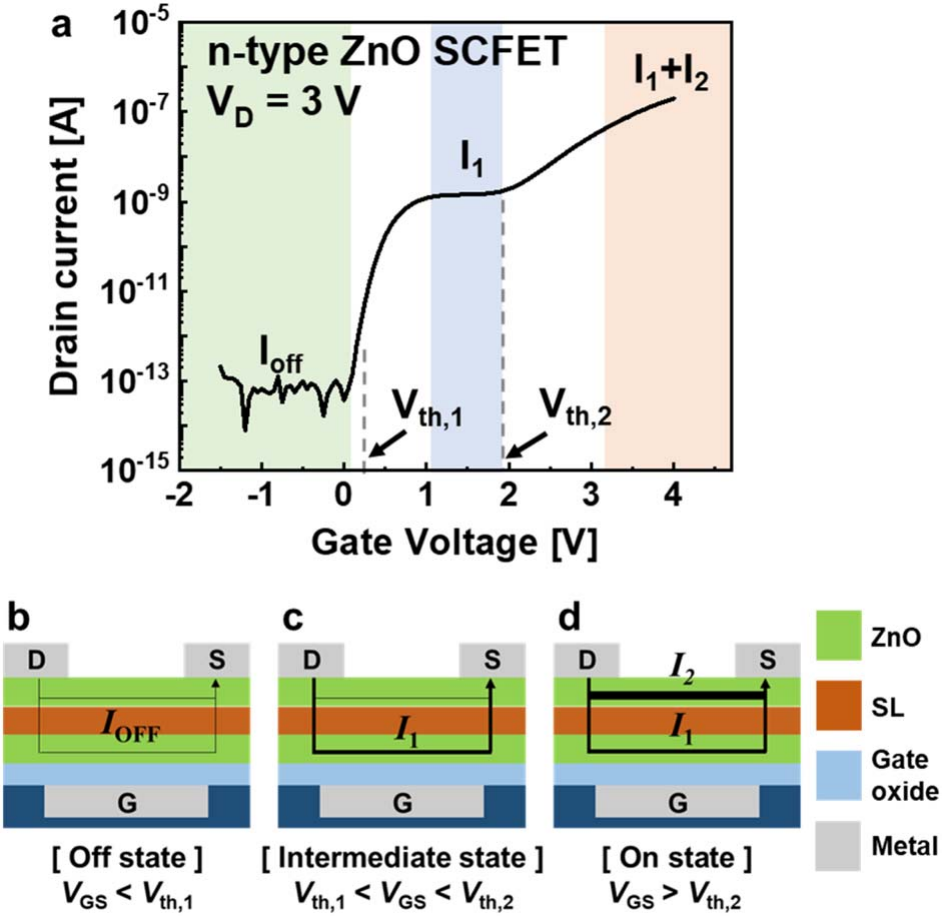
(a) Experimental *I*_D_–*V*_G_ characteristics of the n-type ZnO SCFET. The current transport mechanism for ternary operation of n-type SCFETs at (b) off state, (c) intermediate state, and (d) on state.

The *I*_D_–*V*_G_ curve of an n-type SCFET exhibits two distinct threshold voltages, which define three operational states (Fig. 2a). When the gate voltage (*V*_GS_) is below the first threshold voltage, the device is in an off-state (green area) where only off-current (*I*_OFF_) flows through the channels. Upon exceeding the first threshold voltage (*V*_th,1_), the current increases to an intermediate state level (blue area), where the current is saturated to a specific value (*I*_1_) determined by the first ZnO layer. As the gate bias increases further and reaches the second threshold voltage (*V*_th,2_), the current begins to increase once more, entering the on-current region (red area). The primary current paths passing through two channel layers, yielding the stepwise ternary *I*–*V* curve, are schematically shown in Fig. 2b–d. Two channels are turned on sequentially because of the differences in the threshold voltage. First, when *V*_GS_ is lower than *V*_th,1_, the device remains in the off state, as illustrated in Fig. 2b. As *V*_GS_ increases above *V*_th,1_, the conductivity of the first layer increases, as shown in Fig. 2c; however, the current level saturates to *I*_1_ because of the constant carrier concentration in the conduction band.^25^ Finally, when *V*_GS_ exceeds *V*_th,2_, the second layer becomes conductive, and the device has the total current *I*_1_ + *I*_2_, as shown in Fig. 2d. Further analysis of the carrier transport mechanism can be found in ref. 26.

**Fig. 3:**
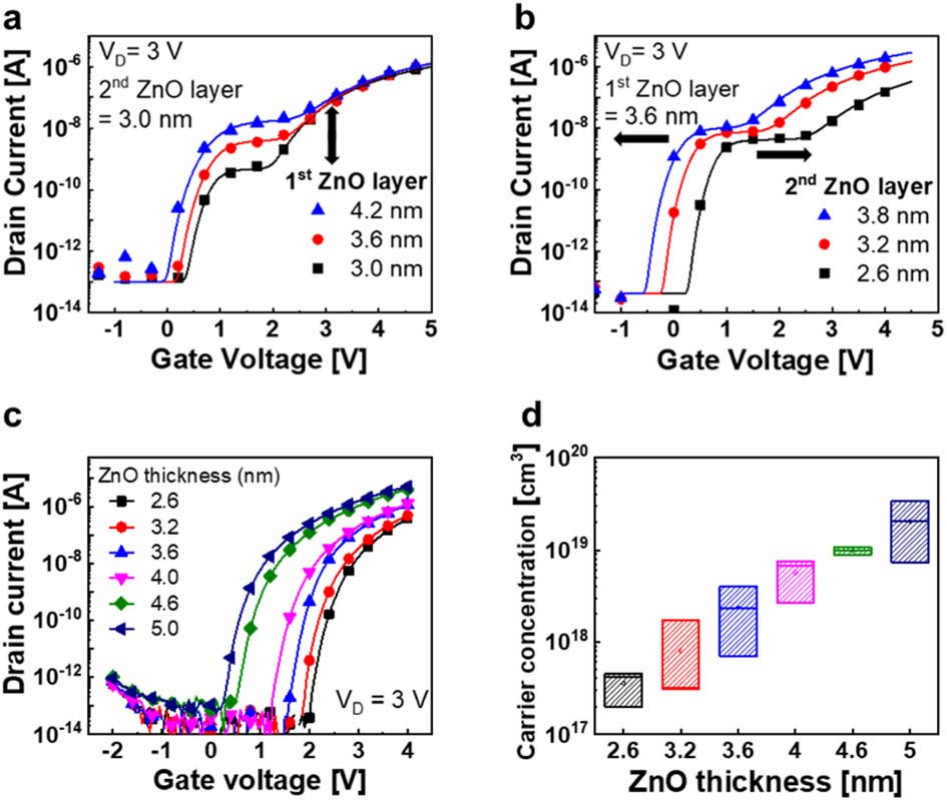
Experimental and simulated *I*_D_–*V*_G_ characteristics of the SCFET modulated using the thickness of the (a) first and (b) second ZnO layers (symbols: experimental data and solid lines: simulated data). (c) *I*_D_–*V*_G_ characteristics of ZnO FETs modulated using the ZnO channel thickness. (d) Carrier concentration of ZnO channels with varied thickness. All direct current (DC) measurements were performed using a semiconductor parameter analyzer. The carrier concentration was measured using an SKPM.

Fig. 3a and b illustrate the *I*_D_–*V*_G_ curves of the n-type SCFET modulated as functions of the thicknesses of the first and second ZnO channel layers (*t*_1_ and *t*_2_), respectively. The symbols represent the experimental data, and the solid lines indicate the simulation results obtained using the semi-empirical SCFET model explained below. As *t*_1_ increases, *I*_1_ increases, and only *V*_th,1_ shifts slightly. These experimental results can be explained by the change in the carrier concentration of each channel layer, which is affected by the thickness and gate bias.^26,29^ To support this claim, the electrical characteristics of single channel ZnO FETs and the variation in the carrier concentration with channel thickness are shown in Fig. 3c and d. By contrast, when *t*_2_ increases, the entire *I*_D_–*V*_G_ curve shifts to the left in each case. According to the simple device model described in Fig. 2, *t*_2_ should affect only *V*_th,2_. However, the experimental results show that *V*_th,1_ and *V*_th,2_ move simultaneously as *t*_2_ changes. Even though the process temperature is extremely low, the deposition process for the 2nd ZnO layer is a few tens of minutes (288 s for one ALD cycle). Thus, the extended thermal cycle appears to have some impact on *V*_th1_.

The thickness of the Al-DMP/Al_2_O_3_ SL also affected the SCFET characteristics (Fig. S1, ESI†). However, when the thickness exceeds 10 nm, the *V*_th,1_ shifts to the negative side, which is too severe to be used for logic circuit design. Additionally, the bidirectional *I*–*V* sweep showed a small hysteresis (<80 mV) in the subthreshold region (Fig. S2, ESI†). Therefore, the SL thickness changes and hysteresis were not considered in the subsequent modeling. All electrical characteristics data were measured using a semiconductor parameter analyzer (Keithley 4200) at 25 °C in ambient air. The carrier concentration of ZnO FETs was measured using scanning Kelvin probe microscopy (SKPM).

To simulate the applicability of SCFETs, a SPICE model was developed using the experimental parameters as summarized in Table 1. In this work, compared to the previously reported SCFET,^26^ a thinner *t*_1_ (3.0 nm) was used to obtain the extremely low *I*_1_ for low power consumption. In addition, the *t*_2_ range was expanded from 2.6 to 3.8 nm to model more negatively shifted *I*–*V* curves. These data were used to design a model for the low-power circuits discussed in the next section. Essentially, our model emulates a ternary device by combining two FETs with different threshold voltages. The model for each FET is expressed by the following eqn (1):^30,31^1
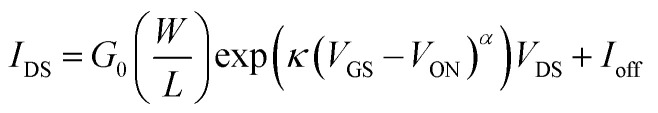
where *G*_0_ is a parameter related to the mobility; *W* and *L* represent the width and length, respectively, of the channel; *κ* and *α* are empirical parameters for compact modeling;^30,31^*V*_on_ represents the turn-on voltage at which the drain current begins to increase; and *I*_off_ represents the off-current. For the saturation region, *V*_DS_ can be replaced with (*V*_GS_ – *V*_on_). The difference in *V*_on_ between the two FETs defines the intermediate state. *V*_on,1_ is a function of *t*_1_ and *t*_2_, *V*_on,2_ is a function of *t*_2_, and the intermediate-state current *I*_1_ is primarily affected by *t*_1_, according to the experimental results. Detailed correlation and equations are shown in Fig. S3, ESI.† This model matches the measured data with over 95% accuracy, as shown in Fig. 3a and b.

**Table 1:** Experimental parameters used in the SCFET model

	EOT (nm)	*L*_ch_ (μm)	*W*_ch_ (μm)	*t*_1_ (nm)	*t*_2_ (nm)	*I*_1_ (nA)
25	5.6	60	24	2.8	2.8	3.0
26	9.0	12	24	3.2–5.0	2.6–3.6	1–100
This work	8.2	12	16	3.0–4.2	2.6–3.8	0.4–12

**Fig. 4:**
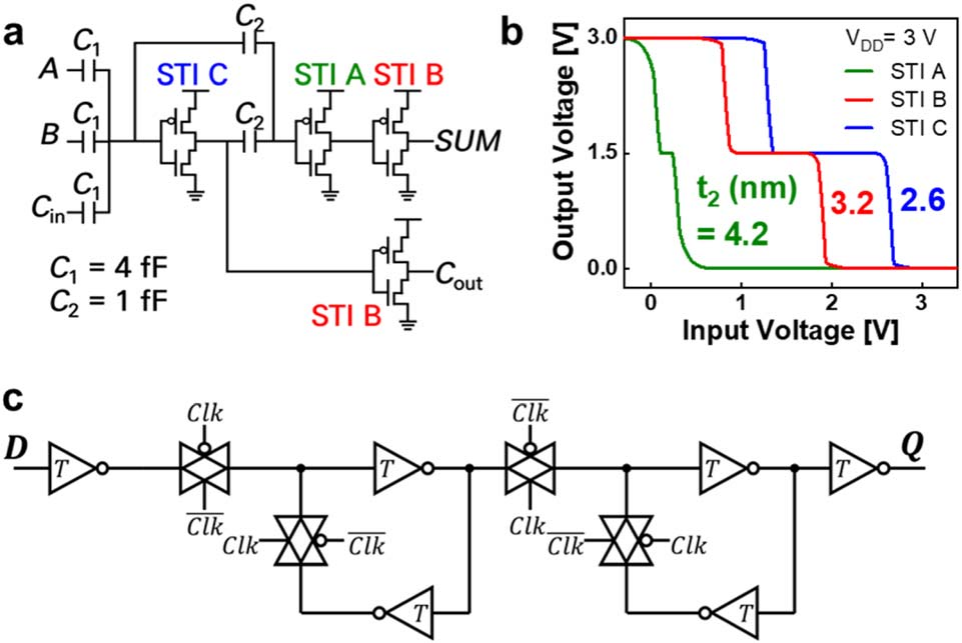
(a) Circuit diagram of a ternary full adder using capacitors. (b) Simulated transfer characteristics of standard ternary inverters (STIs) A, B, and C. (c) Circuit diagram of the ternary flip-flop. The inverter symbol with a capital “*T*” represents STI B.

We simulated the functionality and performance of the TSA to evaluate the feasibility of the SCFET as unit devices for a synchronous ternary logic system. The TSA design requires one TFA and a ternary flip-flop (TFF). Fig. 4a shows the circuit diagram of the TFA designed with capacitors and three types of STIs, with only 13 devices, including the capacitors. This number is even smaller than that of a binary full adder (42 devices) designed with silicon CMOS transistors. The simulated transfer characteristics of the three different STIs—A, B, and C—are shown in Fig. 4b. For these three STIs, the *t*_2_ values were adjusted to 4.2, 3.2, and 2.6 nm, respectively. A p-type SCFET model used in STIs was also developed using the experimental data from a dinaphtho[2,3-*b*:2′,3′-*f*]thieno[3,2-*b*]thiophene (DNTT)-based SCFET^27^ (Fig. S4, ESI†), but the thickness dependence was assumed to be the same as that of n-type SCFETs. The p-type SCFET needs further study with much thinner p-type semiconductor materials.

The TFF was designed as a positive edge-triggered master-slave D flip-flop using STI B and transmission gates, as shown in Fig. 4c. The TFF captures a ternary value using a positive feedback loop of back-to-back STIs at a positive edge of the binary clock signal using binary FET-based transmission gates. The proposed TFF was designed using 12 SCFETs and eight binary FETs.

**Fig. 5:**
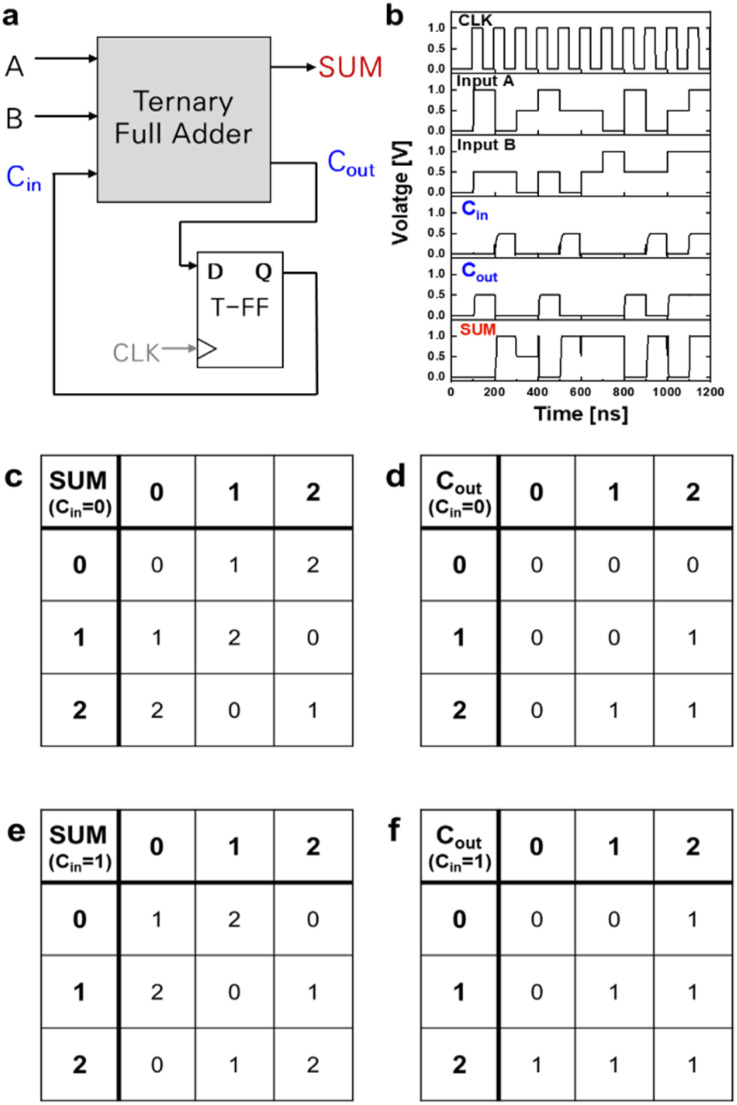
(a) Schematic of the TSA. (b) Simulated transient responses of the TSA; simulation was performed using Synopsis HSPICE. Truth tables of (c) SUM and (d) *C*_out_ when *C*_in_ = 0 and (e) SUM and (f) *C*_out_ when *C*_in_ = 1.

Finally, the TSA was designed by combining the TFA and TFF circuits as shown in Fig. 5a. The transient responses of the TSA circuit were investigated using Synopsys HSPICE with a 0.01 GHz operation frequency and 1 ns transient time. The fanout load capacitance of each STI was 1 fF, and the *C*_1_ and *C*_2_ values were 4 and 1 fF, respectively. For this simulation, the device parameters used for the SCFET model were adjusted to *V*_DD_ = 1 V,^32^ effective oxide thickness (EOT) = 1 nm, and channel length = 100 nm, for reasonable comparison with previous studies. The thickness of the ZnO layer was varied within a reasonable range, *t*_1_ = 3.0–4.2 nm and *t*_2_ = 2.6–4.2 nm, to design the different STIs shown in Fig. 4a. As illustrated in Fig. 5b, when specific input signal patterns (*A*, *B*) were inserted, the output carry signal (*C*_out_) was successfully fed again to the input carry (*C*_in_) node after one positive edge cycle through the TFF. Consequently, a serial addition operation that matched the clock signal was verified. The truth tables of the TSA are shown in Fig. 5c–f.

To compare the energy efficiencies of SCFET-based TSAs with that of the TSA designed with CNTFETs,^33^ the average power consumption and worst propagation delay were simulated (Table 2). In the case of SCFETs, the TSA performance was examined by varying *t*_1_, which controls the level of *I*_1_. *t*_1_ significantly influences the power and delay because most of the power consumption and the worst delay are determined by the charging and discharging steps of the half-*V*_DD_ state, which are related to *I*_1_. Consequently, as *t*_1_ decreases, the PDP improves faster than the delay degradation. The tradeoff between power and delay as a function of *t*_1_ is shown in Fig. 6. Under the minimal *I*_1_ (1/100) condition, the TSA consumed one-tenth the power of the CNTFET-based TSA and exhibited one-third of the PDP. Although our proposed design still requires SCFETs with three different channel thicknesses, it is still more practical than the fabrication of CNTFETs with six different CNT diameters. Thus, SCFETs are promising alternatives to CNTFETs in ternary circuit applications.

**Table 2:** Power and delay of TSAs designed with SCFETs and CNTFETs

	# Of the devices	Normalized *I*_1_	Power (μW)	Delay (ns)	PDP (fJ)
SCFET (this work)	33	1	13.83	6.71	92.80
		1/3	5.188	7.01	36.37
		1/10	1.677	8.52	14.29
		1/30	0.5771	12.9	7.44
		1/100	0.2108	33.9	7.15
CNTFET^31^	55	x	3.079	6.54	20.11

**Fig. 6:**
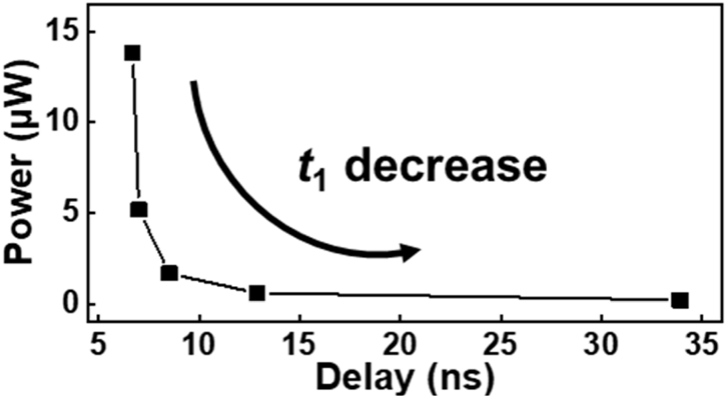
Simulation result showing the tradeoff relationship between power and delay with a reduction in the first ZnO layer thickness *t*_1_.

## Conclusions

We demonstrated a low-power TSA using SCFET technology that features a minimal device count of 33 and a PDP of approximately 7 fJ at *V*_DD_ = 1 V and EOT = 1 nm. These performance results are highly competitive with those of an ideal CNTFET-based circuit. SCFETs can be a practical solution to realize the various ternary logic circuits proposed using multi-diameter CNTFETs, primarily because the fabrication process is more practical owing to the low-thermal-budget deposition process, excellent thickness controllability, and wafer-scale process compatibility. Considering that it has the lowest PDP among alternative technologies, the ternary circuit using SCFETs is a promising technology for extreme low-power applications.

## Data availability

All data generated or analysed during this study are included in this published article [and its ESI†].

## Author contributions

K. K. and S. K. contributed equally to this work. K. K., S. K., and B. H. L. conceived the idea and designed the experiments and simulation. K. K. and S.-Y. K. executed experiments and performed material and electrical characterization studies. S. K. contributed to the circuit design and simulation. All authors were involved in discussions.

## Conflicts of interest

There are no conflicts to declare.

## Supplementary Material

NA-OLF-D5NA00045A-s001
