# Supplementary material for: An area and power efficient ternary serial adder using phase composite ZnO stack channel FETs
Source: Nanoscale Adv. 2025 Apr 8;7(11):3508–13. doi: 10.1039/d5na00045a (PMC12038365; doi:10.1039/d5na00045a)
Supplement: NA-007-D5NA00045A-s001 [file NA-007-D5NA00045A-s001.pdf]

Supplementary Information (SI) for Nanoscale Advances.
This journal is © The Royal Society of Chemistry 2025

Supporting Information

Area and Power Efficient Ternary Serial Adder Using Phase Composite ZnO Stack Channel FETs

*Kiyung Kim, Sunmean Kim, So-Young Kim, Yongsu Lee, Hae-Won Lee, Seokhyeong Kang, and Byoung Hun Lee**

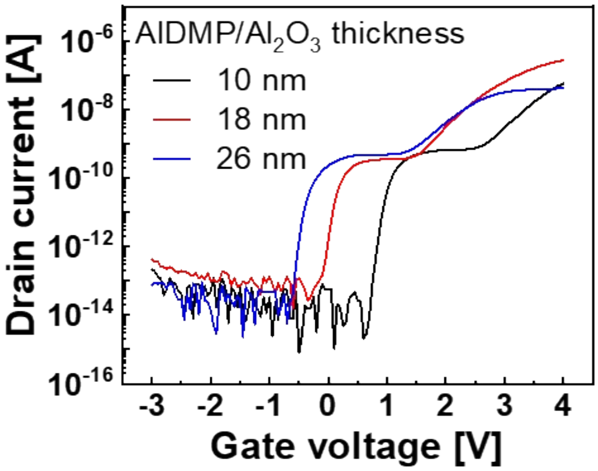

Fig. S1. Effect of the Al-DMP/Al_2O_3 separation layer thickness on the SCFET characteristics.

The thickness of the Al-DMP/Al_2O_3 SL significantly affected the characteristics of the SCFET. As the thickness increased, $V_{th,1}$ rapidly shifted towards negative side, and it became difficult to maintain the enhancement mode operation.

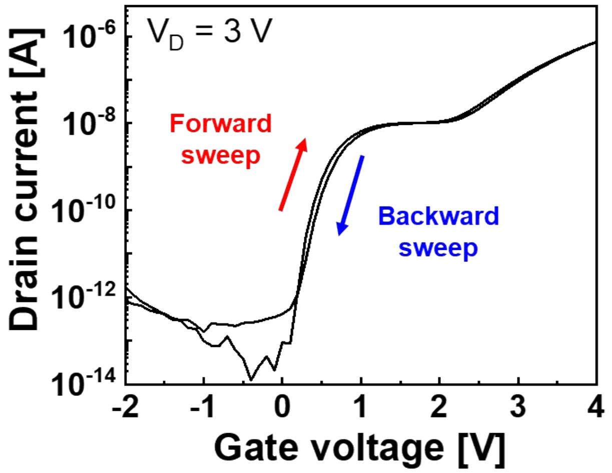

Fig. S2. Experimental I_D–V_G characteristics of the ZnO SCFETs obtained from bidirectional I–V sweep. The first and second ZnO layer thicknesses were 3.6 nm and 3.2 nm, respectively.

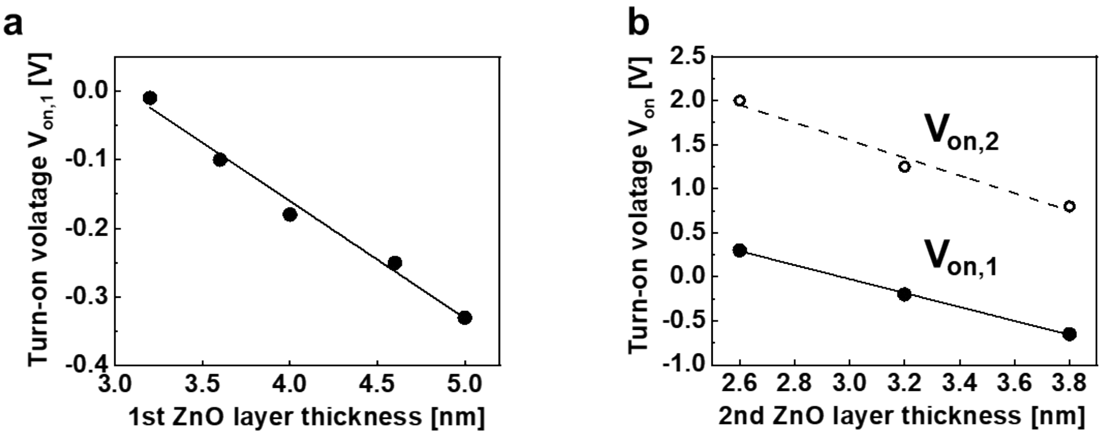

Fig. S3. a) Correlation between the thickness of the first ZnO layer and turn-on voltage 1. b) Correlation between the thickness of the second ZnO layer and turn-on voltages 1 and 2.

$$V_{on,1} = -0.1703\, t_1 - 0.792\, t_2 + 2.87 \qquad (1)$$

$$V_{on,2} = -\, t_2 + 4.55 \qquad (2)$$

Equation (1), extracted from the experimental data, expresses the correlation between the turn-on voltage 1 ($V_{on,1}$) and thicknesses of the first and second ZnO layers.

Further, Equation (2), extracted from the experimental data, expresses the correlation between $V_{on,2}$ and the thickness of the second ZnO layer.

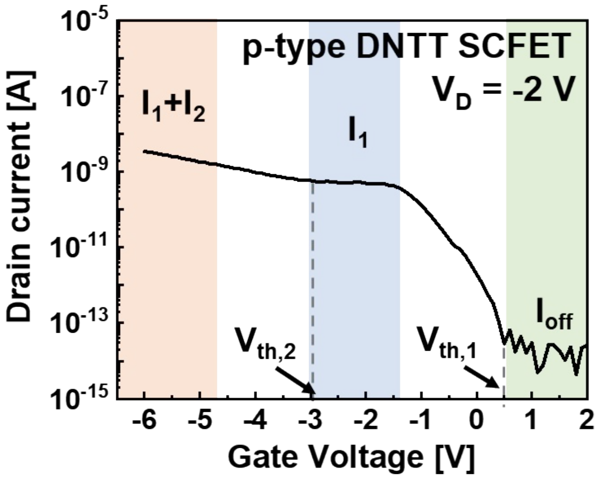

Fig. S4. Experimental I_D–V_G characteristics of p-type dinaphtho[2,3-b:2’,3’-f]thieno[3,2-b]thiophene (DNTT) SCFET.
